# Circular Economy and Supply Chains: Definitions, Conceptualizations, and Research Agenda of the Circular Supply Chain Framework

**DOI:** 10.1007/s43615-022-00172-y

**Published:** 2022-05-13

**Authors:** Laura Montag

**Affiliations:** Chair of Production Management, Ruhr University Bochum, Universitätsstraße 150, 44801 Bochum, Germany

**Keywords:** M11, Q56, Circular economy, Supply chain management, Circular supply chain, Systematic literature review

## Abstract

**Objective:**

Circular supply chain management (CSCM) incorporates circular thinking — based on the circular economy paradigm — into supply chain management. In the last 5 years, this emerging research field has developed at a rapid pace and, as a result, has attracted great interest from researchers, policy-makers, and practitioners. As there are few studies on the theoretical conceptualization of the circular supply chain (CSC), especially on its definition, this paper aims to fill this gap and to provide conceptual transparency for the CSC framework. The main research question is “What are the current understandings among scholars of the CSC concept and CSCM framework?”.

**Method:**

To answer this question, a systematic literature review was conducted based on the Web of Science and Scopus databases. This was followed by a bibliometric analysis using VOSviewer and a comprehensive content analysis of the literature.

**Results:**

The bibliometric analysis provided an overview of CSC evolution and identified three temporal, thematic clusters. The content analysis identified 127 articles that explicitly mention the term CSC(M). Of these, seventeen articles provide explicit definitions that were thoroughly analyzed and categorized. Following this, six archetypal elements of the CSC and four propositions on the CSC’s uniqueness were formulated.

**Conclusion:**

The CSC research field is evolving rapidly. Its differentiation from other sustainability-related fields is sometimes not clear, and definitions and conceptualizations vary in detail, scope, and focus.

**Contributions:**

This study contributes to the CSC literature and provides transparency for the conceptualization and understanding of CSC. For both theory and practice, an agenda for future research opportunities is identified, which supports the further development of this research field.

## Introduction

For the last two decades, sustainability has been an emerging topic of interest for science, business, and politics. In particular, the incorporation of sustainability-related topics into supply chain management (SCM) has evolved toward a fast-growing and high-volume research stream [1]. This evolution has led to various supply chain–related sustainability frameworks such as green supply chains, closed-loop supply chains, reverse supply chains, sustainable supply chains (SSC) and — most recently — circular supply chains (CSCs) [2–5]. The latter integrates circular thinking based on the circular economy (CE) framework — concerning issues such as recycling, reuse, and a zero waste ideal — into the management of supply chains [6, 7].

The CE framework is an economic model that is restorative and regenerative by design [8]. By definition, the CE aims to replace the end-of-life concept by slowing, closing, and narrowing material as well as energy loops [9]. Within a CE, waste itself becomes input, and products, components, and materials are kept at their highest utility level [8]. In the last 5 to 10 years, interest in this concept has increased significantly, and scientific research has grown immensely [10]. However, as the CE concept has antecedents in many different historical, economic, and ecological fields [11], its conceptualization is proving difficult, leading to various definitions, scopes, and narratives [10]. According to some scholars, the fact that the CE framework is trending and rapidly growing but at the same time still remains open and more divergent than convergent could lead to a collapse, diffusion, or falling behind of its attributed potential [10, 12, 13]. To elevate the CE to a new (sustainability) paradigm, a unified perspective is needed [14].

Despite the limited conceptual consensus regarding the CE framework, especially its relationship to sustainability itself, the SCM domain adopted a circular philosophy, shifting the focus to the newly emerging research field of circular supply chain management (CSCM) [3, 4]. From a sustainability viewpoint, CE strategies could support efforts to holistically integrate the economic, environmental, and social dimensions along the supply chain [4, 15]. However, the conceptualization of the CSC is still at a nascent and early stage. In particular, clear boundaries between other sustainability-related SCM frameworks remain ambiguous, and therefore, the current knowledge base on CSCs is fragmented [16].

Within the last 5 years, several review papers on the CSCM framework have been published. Table 1 presents an overview of the most important studies and their main topics and research purposes. Furthermore, this table compares and positions the present work in relation to these existing reviews. With the exception of study [7] — which collected understandings of the CSC framework but did not explicitly focus on conceptualization and a concrete definition — none of the studies in the literature review seems to explicitly focus on CSC and CSCM definitions and thus on understanding the CSC as a differentiated concept. Based on the reviewed literature and the findings of this study, it can be concluded that — to the best of the author’s knowledge — no study has comprehensively and systematically investigated CSC definitions despite both the academic and practical relevance for CSC operationalization [10].

**Table 1:** Previous CSCM review papers (partially adapted from Zhang et al. 86)

Authors	Year	Framework of analysis	Review types and databases	Coverage	Outcomes
Masi et al. [16]	2017	CE on meso-level of SCs	• SyLR • WoS, Scopus, ProQuest	77 articles	• CE definitions • CE drivers for SCs • CE inhibitors and enablers for SCs
Batista et al. [2]	2018	CSC	• Content-based SyLR • EBSCO, ProQuest	49 articles	• Five propositions on the CSC archetype • New CSC definition
De Angelis et al. [17]	2018	SCM + CECSC	• SyLR • not stated	84 articles	• Framework of CSC propositions •Key SC challenges
Geissdoerfer et al. [18]	2018	BM+SCs for CE	• LR+four case studies • not stated	not stated	• Framework on CSCs + BMs for SD •Definition of CSC + CSCM
Farooque et al. [3]	2019	CSCM	• StLR • Scopus	261 articles	• Definition of CSCM • CE integration at different SC levels and functions •Future research agenda
Gonzalez-Sanchez et al. [7]	2020	CSC	• SyLR • Scopus	50 articles	• Definition of CSC + CSCM • Four propositions and dimensions of the CSC
Lahane et al. [4]	2020	CSCM	• SyLR • Scopus	125 articles	• Evaluation against 13 predefined categories • Future research directions
Cerqueira-Streit et al. [19]	2021	SSCM + CE	• Integrative LR • WoS+Scopus	37 articles	• Evolution of publications, identification of relationship networks and themes • Future research suggestions
Lengyel et al. [20]	2021	CSCM	• SyLR • Scopus	6095 articles	•Bibliographic analysis (VOSviewer) •Evolution of scientific documents
Zhang et al. [21]	2021	CE in practice + academic CSCM	• Comparative review • Scopus	60 cases + 124 articles	• Multidimensional CSCM framework • Gaps in research + practice • Future research directions
This current paper	2022	CE, SC + sustainability > CSC	• SyLR • WoS + Scopus	127 articles	• Collection of CSC(M) definitions • Evolution of CSC(M) research field • Four propositions on CSC’s uniqueness

*BM* business model, *CE* circular economy, *CSC* circular supply chain, *LR* literature review, *SC* supply chain, *SD* sustainable development, *StLR* structured literature review, *SyLR* systematic literature review, *WoS* Web of Science

To support the conceptualization and understanding of the CSC at this research stage, this review mainly aims to provide transparency for the CSC framework, filling the gap in the literature bridging SCM, sustainability, and CE [17]. After some years of the existence of the term “circular supply chain,” this review concentrates on its conceptualization within the research field by synthesizing the outcomes of previous research papers on the CSC. This paper follows recent calls by [2–4], and [19] to analyze supply chains in light of the CE since there is momentum for progressing the theory, especially in terms of its conceptualization. Consequently, the main research question of this paper is as follows:

- RQ: What are the current understandings among scholars of the CSC concept and CSCM framework?

To properly address this research question, three sub-questions are formulated that serve as guidance throughout this research paper:

- RQ1: What are the main research streams and core topics within the research field?
- RQ2: What are the definitions, conceptualizations, and understandings within the research field?
- RQ3: What makes the CSC concept unique, and thus, what differentiates it from other concepts?

In response to the research questions, a systematic literature review is conducted by applying the search terms “supply chain,” “circular economy,” and “sustain*” to the two most often used databases, Web of Science (WOS), and Scopus [22]. The main objective of this search is to examine CSC definitions and conceptualizations to provide comprehensive and systematic transparency on the current state of knowledge and understanding of CSCs. Through a systematic literature review as the study’s adopted methodology [23], a thorough and in-depth exploration and analysis is facilitated. After a filtering process, an overall final sample of 127 papers is identified. This sample builds the basis to answer the previously formulated research questions. The literature search was updated (1st of March 2022) and thus reflects the very recent state of the research in the CSCM field.

The remainder of this paper is organized as follows: “Conceptual Background” provides a brief overview of the conceptual background, focusing on the three research streams of SCM, sustainability, and the CE. “Methodology” explains in detail the methodology behind the systematic literature review and explains how the final article sample was compiled. The fourth section contains the results of the systematic literature review and the following discussion on CSC uniqueness. First, a general overview of the literature sample is given before RQ1 is addressed by presenting the bibliometric results from VOSviewer. The qualitative results obtained from the content-based analysis are discussed in the subsequent section and answer to RQ2. RQ3 is addressed in the subsequent section, in which four CSC propositions on its uniqueness are presented. Finally, “Conclusion” summarizes the main theoretical and practical contributions of this research paper, elaborates on some limitations, and suggests future research directions for the CSC research framework.

## Conceptual Background

Since the purpose of this literature review is to analyze the understanding and interrelationships of traditional, sustainable, and circular supply chains, it is important to briefly establish the conceptual background of the studied concepts and their terms on which the following review section is based. Therefore, the first part of this section focuses on supply chains and their management; the second part briefly summarizes the evolution of the sustainability influence on the SCM research field; and, finally, the background and different definitions of the CE concept are presented.

### Supply Chains and Supply Chain Management

With increased competition in globalized markets, higher customer expectations, and shorter product-life cycles, organizations require the effective management of their processes, information and relationships, which often go beyond their national boundaries [24]. In this competitive environment, management of these multiple relationships across the supply chain is necessary [25].

The terms supply chain and SCM first appeared in the early 1980s in the USA and were coined by practitioners and consultants [26]. Although they gained immense attention in practice, the theory from academia developed only later in the 1980s and early 1990s [27, 28]. Since then, the literature on the SCM framework has grown enormously, and various fields have developed within this research stream, e.g., operations management, purchasing, and supply or organizational theory [29]. While in the beginning, the term supply chain management might have often been misunderstood as only the logistics outside of the company, the conceptualization changed [25]. To stay competitive and successful, supply chain decisions — and therefore SCM — play a significant role in every organization [30].

For successful management, it is important to understand which entities are part of the supply chain. In Handfield and Nichols’ definition, all parties involved in “activities associated with the flow and transformation of goods from raw materials stage (extraction) through to the end user, as well as the associated information flows” [27] belong to a supply chain. Similarly, Chopra and Meindl (2016) define that a “supply chain consists of all parties involved, directly or indirectly, in fulfilling a customer request” [30]. There are a variety of stages that can be involved in a typical supply chain and include suppliers, manufacturers, distributors, retailers and, of course, (end-)consumers [30]. Although the term chain suggests that there are only one-to-one relationships, a supply chain is actually more accurately described as a network since there are multiple businesses, parties, and therefore relationships involved [31].

The actual management of the supply chain aims to efficiently and strategically integrate all upstream and downstream processes, operations, (information) flows, and relationships in a way that adds value for customers and all supply chain stakeholders involved [24, 27, 31].

### Supply Chains and Sustainability

In 1987, the World Commission on Environment and Development released the Brundtland Report introducing the concept of sustainable development [32], which was a milestone for future sustainability research. Not only does the UN commission’s definition play a crucial role in sustainability research to this day [6], but it has also led to a remarkable increase in publications dealing with sustainability-related issues [1]. Among the various research areas of SCM that have developed over the 30 years of its existence, SSCM is one of the most dynamic and productive research streams [1].

Within the UN World Commission’s report, sustainable development is defined in an inter- and intragenerational context. Development can only be sustainable if others — within this generation and all future generations to come — are not compromised in their (future) needs [32]. While some criticize the vagueness and room for interpretation left by this definition [9, 33, 34], it should also be acknowledged that this definition is used as a common framework that can be appropriately extended to different contexts and applications.

A comprehensive concept in the context of business sustainability was introduced with John Elkington’s 3P formulation together with the triple bottom line, which he developed shortly thereafter [35, 36]. The 3Ps represent the people, the planet, and the profit for which each business organization should account. Instead of only focusing on the traditional financial (bottom) line, companies need to account for their environmental impacts (the planet’s bottom line) as well as their social impacts (humanity’s bottom line). In the context of business sustainability, the three-pillar perspective or the three-dimensional sustainability approach became synonyms that were widely used in academia and practice [1].

In the SCM framework — due to the research interest and the resulting scores of publications — quite a few definitions of sustainability exist, most of them directly in the context of supply chains. An early literature review on the SSCM framework was conducted by Seuring and Müller in 2008. The researchers considered 191 papers published between 1994 and 2007 and provided a now well-known, widely accepted definition of SSCM. This definition and a few other, illustrative definitions are presented in Table 2.

**Table 2:** Illustrative definitions of SSCM

Author(s)	Definition sustainable supply chain (management)
Seuring, Müller [37]	[W]e define sustainable supply chain management as the management of material, information and capital flows as well as cooperation among companies along the supply chain while taking goals from all three dimensions of sustainable development, i.e., economic, environmental and social, into account which are derived from customer and stakeholder requirements. [p. 1700]
Carter, Rogers [38]	[W]e define SSCM as the strategic, transparent integration and achievement of an organization’s social, environmental, and economic goals in the systemic coordination of key interorganizational business processes for improving the long-term economic performance of the individual company and its supply chains. [p. 368]
Ahi, Searcy [39]	The creation of coordinated supply chains through the voluntary integration of economic, environmental, and social considerations with key inter-organizational business systems designed to efficiently and effectively manage the material, information, and capital flows associated with the procurement, production, and distribution of products or services in order to meet stakeholder requirements and improve the profitability, competitiveness, and resilience of the organization over the short- and long-term. [p. 339]

In the SCM literature, a number of other frameworks — such as green supply chains [39], closed-loop supply chains [40], and social/ethical supply chains [37] — emerged within the sustainability narrative. A lack of conceptualization and especially distinction between these concepts led to the interchangeable and synonymous use of terms in the scientific literature [2]. As SSCs can be perceived as extensions of green, social or closed-loop supply chains [39], and the focus within this review is on three-dimensional sustainability in the context of supply chains, no further input on these terms is provided.

Although these concepts differ in their degree of sustainability orientation integrated in the supply chain, none of them fully and systematically integrate circular principles in the context of a CE ideal [3]. The next section provides an overview of the CE paradigm, its development, definition, and key characteristics.

### Circular Economy

The idea of circular flows and systems is not as new as it is perceived to be at the moment. Due to the increasing interest in CE in the last 5 to 10 years, it appears to be a promising and rapidly growing research field [41–43], but the remote origins of the concept actually date back to Boulding in 1966 [44, 45]. Although Boulding did employ the exact term “CE,” he refers to “cyclical ecological systems” without which human survival on earth cannot be sustained in the long run [46].

Building on Boulding’s ideas, an introduction of the CE term is mostly associated with Pearce and Turner in the late 1980s and early 1990s [45, 47]. Based on investigations on the impacts of linear and open-ended systems, they argued that a traditional linear economy must be replaced by a cyclical system in which waste becomes input for the system [45, 47–49].

In regard to the roots of the actual concept of CE and its application in practice, one must consider several related concepts, frameworks, and research streams that are more or less closely associated with the CE such as cradle-to-cradle, industrial ecology, the performance economy, the blue economy, and regenerative design. For further in-depth studies on these schools of thought and their relationship to the CE concept, see, for example, research from Ghisellini, Cialani, Ulgiati [47], Geisendorf, Pietrulla [45], Homrich et al. [41] and Merli, Preziosi, Acampora [42].

As the CE concept has antecedents in many different historical, economic and ecological fields [11], it has proven difficult to obtain a cross-disciplinary understanding, leading to various definitions, scopes, and CE narratives in research and practice [10]. There is agreement among researchers that there is a lack of consensus on the definition of CE, although it is of great importance for both academia and practice [13]. Some studies have been undertaken to gather the most important definitions and conceptualizations to create transparency for this emerging research field; see, e.g., Kirchherr, Reike, Hekkert [10], who collected 114 different definitions of the CE framework.

According to some scholars, the fact that employment of the CE concept is trending and rapidly growing but at the same time remains open and more divergent than convergent could lead to a collapse, diffusion, or falling behind of its attributed potential [10, 12, 13]. To elevate the CE framework to a new (sustainability) paradigm, a unified perspective is needed [14].

Table 3 summarizes some of the most prominent definitions currently being used, drawn from academia, politics, and the UK-based charity the Ellen MacArthur Foundation, a non-governmental flagship organization promoting the CE and its concept. Again, this collection should be understood as a representative presentation of definitions collected based on a literature scan rather than a systematic literature search. Therefore, the chronological collection is not comprehensive but rather illustrative.

**Table 3:** Illustrative definitions of CE

Author(s)	Explicit definition of CE
Ellen MacArthur Foundation [8]	The [CE] concept is characterised, more than defined, as an economy that is restorative and regenerative by design and aims to keep products, components, and materials at their highest utility and value at all times, distinguishing between technical and biological cycles. It is conceived as a continuous positive development cycle that preserves and enhances natural capital, optimises resource yields, and minimizes system risks by managing finite stocks and renewable flows. It works effectively at every scale. This economic model seeks to ultimately decouple global economic development from finite resource consumption. [p. 5]
European Commission [50]	In a circular economy the value of products and materials is maintained for as long as possible; waste and resource use are minimised, and resources are kept within the economy when a product has reached the end of its life, to be used again and again to create further value. [3^rd^ paragraph]
European Parliamentary Research Service [51]	A production and consumption model which involves reusing, repairing, refurbishing and recycling existing materials and products to keep materials within the economy wherever possible. A circular economy implies that waste will itself become a resource, consequently minimising the actual amount of waste. It is generally opposed to a traditional, linear economic model, which is based on a 'take-make-consume-throw away' pattern. [paragraph Definitions]
Kirchherr, Reike, Hekkert [10]	We defined CE […] as an economic system that replaces the ‘end-of-life’ concept with reducing, alternatively reusing, recycling and recovering materials in production/distribution and consumption processes. It operates at the micro level (products, companies, consumers), meso level (eco-industrial parks) and macro level (city, region, nation and beyond), with the aim to accomplish sustainable development, thus simultaneously creating environmental quality, economic prosperity and social equity, to the benefit of current and future generations. It is enabled by novel business models and responsible consumers. [p. 229]
Geissdoerfer et al. [9]	[W]e define the Circular Economy as a regenerative system in which resource input and waste, emission, and energy leakage are minimized by slowing, closing, and narrowing material and energy loops. This can be achieved through long-lasting design, maintenance, repair, reuse, remanufacturing, refurbishing, and recycling. [p. 766]
Murray, Skene, Haynes [11]	The Circular Economy is an economic model wherein planning, resourcing, procurement, production and reprocessing are designed and managed, as both process and output, to maximize ecosystem functioning and human well-being. [p. 377]
Korhonen et al. [52]	CE is a sustainable development initiative with the objective of reducing the societal production-consumption systems' linear material and energy throughput flows by applying materials cycles, renewable and cascade-type energy flows to the linear system. CE promotes high value material cycles alongside more traditional recycling and develops systems approaches to the cooperation of producers, consumers and other societal actors in sustainable development work. [p. 547]
Bressanelli, Perona, Saccani [53]	CE is defined as an economic system restorative and regenerative by design, implemented by one or more supply chain actors through one or more of the four building blocks (circular product design, servitised business models, reverse logistics and enablers) in order to replace the end-of-life concept with reducing, alternatively reusing, recycling and recovering materials in production, distribution and consumption processes, for both technical and biological materials, with the aim to accomplish sustainable development. [p. 4]
Prieto-Sandoval, Jaca, Ormazabal [14]	The circular economy is an economic system that represents a change of paradigm in the way that human society is interrelated with nature and aims to prevent the depletion of resources, close energy and materials loops, and facilitate sustainable development through its implementation at the micro (enterprises and consumers), meso (economic agents integrated in symbiosis) and macro (city, regions and governments) levels. Attaining this circular model requires cyclical and regenerative environmental innovations in the way society legislates, produces and consumes. [p. 610]
Suárez-Eiroa et al. [54]	[C]ircular economy is a regenerative production-consumption system that aims to maintain extraction rates of resources and generation rates of wastes and emissions under suitable values for planetary boundaries, through closing the system, reducing its size and maintaining the resource's value as long as possible within the system, mainly leaning on design and education, and with capacity to be implemented at any scale. [p. 958]

## Methodology

To answer the established research questions, a systematic literature review was conducted. This research framework was adopted from the literature search process proposed by Tranfield, Denyer, Smart [23]. After finalizing the main research objective of this study — to identify and systematize works addressing the CSC framework and to then gather definitions and conceptualizations on the CSC — three phases of planning, searching, and reporting were followed:

- **Planning**: During the planning phase, the basic systematic literature review process was established. This included the definition of the search terms, search field, and time frame as well as language and publication types. As this research aims to capture the current understanding of the CSC framework and thus tries to identify explicit definitions of the CSC(M) term, the search terms “circular,” “supply chain,” and “sustain*” were selected. The scope of the review included (peer-reviewed) journal articles and conference proceedings published in English. The two most commonly used databases, WOS and Scopus, were selected to mitigate the risk of missing important articles for this research [55].
- **Searching**: The literature search itself was conducted at the beginning of March 2022 and retrieved 1109 articles in the WOS and 930 articles in the Scopus database. After deleting 575 duplicates, 1464 articles remained for further analysis. To this end, further quality criteria for inclusion and exclusion were defined. Thus, articles had to interpret the CSC framework as a new concept, understand it as a theoretical framework, apply the CSC framework to a real world problem, or conduct case studies on the CSC. Based on these criteria, 1199 articles were excluded based on their title and keywords, leaving 265 articles for further, in-depth text screening. Next, 147 articles were excluded based on their content and lack of CSC focus. Nine hand-selected publications, which were found via the snowballing technique [9], were included based on their content fit to the CSC framework. Thus, 127 articles were identified as the final sample.
- **Reporting**: The reporting phase comprised two stages, specifically, descriptive or bibliometric analysis and thematic analysis. In the former stage, two main software packages were used: MS Excel and VOSviewer. VOSviewer is a freely available computer program that can be used for constructing and analyzing bibliometric mapping [56]. This bibliometric analysis aims to answer RQ1, which concerns the general body of knowledge and the current research stream within the field of CSCs. The thematic analysis aims to answer RQ2 and RQ3 and therefore uses a content analysis to systemize and identify definitions and conceptualizations of the CSC framework.

**Fig. 1:**
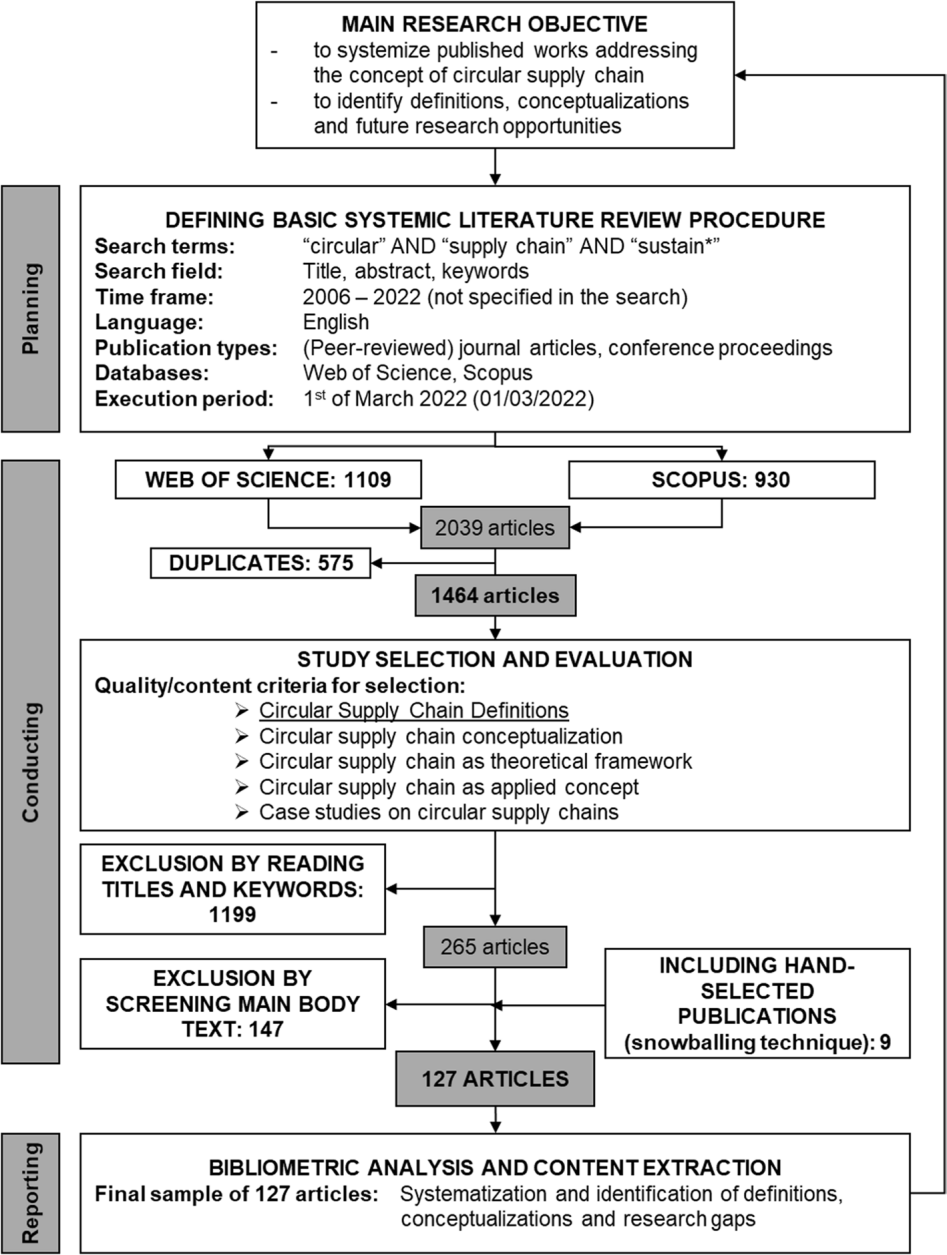
Study selection process

The study selection process as described above is illustrated in Fig. 1 (adopted from Hofmann [57]).

## Results and Discussion

### Sample Overview

**Fig. 2:**
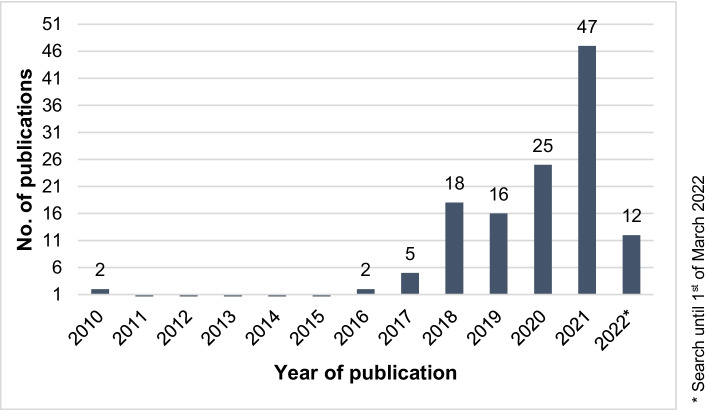
Distribution of publications per year (final sample)

**Fig. 3:**
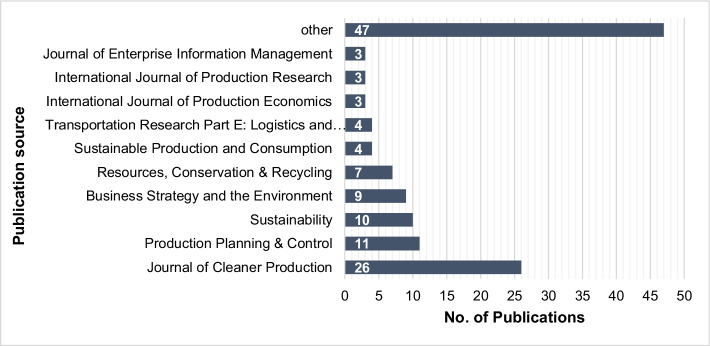
Distribution of publications per journal (final sample)

This section presents the first part of the systematic literature review’s obtained results and aims to give a brief overview of the article sample. As explained in detail in “Methodology”, the final sample consists of 127 research articles. Figures 2 and 3 display these 127 articles and their distribution per publication year (time span from 2010 to 2022) and per publication source.

With the exception of the two publications in 2010 — which are extended conference papers — all other reviewed articles were published from 2016 onward. From 2016/2017 to 2018/2019/2020, a noticeable steady increase in publications was recognized. To date, the peak in publications was reached in 2021, with 47 articles addressing the CSC framework overall. As of March 1, 2022, 12 articles have already been published, indicating that the peak from 2021 may be exceeded this year. This development reflects the growing interest in this research field and stresses the need for a systematization and identification of key articles within the field.

Figure 3 presents the distribution of the final sample across the journals in which they were published. Overall, 51 different journals published contributions to the research field. It was found that 35 journals published only one article on this topic. The *Journal of Cleaner Production*, an international and transdisciplinary journal focusing on sustainability research, tops the list with a 20% share of publications (26). The journals *Production Planning & Control* (11) and *Sustainability* (10) rank second and third, respectively. This distribution indicates that the research field itself is represented from a variety of perspectives and angles and thus has a strong interdisciplinarity.

### Bibliometric Results

To address RQ1 — concerning the research streams and core topics within the research field — bibliometric analysis was carried out using VOSviewer. The software allows for the identification of the leading publications and connections not only in terms of citations and co-authorship but also in terms of the co-occurrence of (author-provided) keywords. The central feature of the applied software is the creation of network maps based on bibliometric data obtained from search databases [56].

To identify current topics and thematic developments within the CSC research field, the co-occurrences of author keywords were mapped. In addition, a temporal overlay function provided by the software was used to connect the keywords with the dates of their source documents. Both the search results from WOS and Scopus were used as a data basis with the aim of obtaining a comprehensive picture of the CSC research field.

**Fig. 4:**
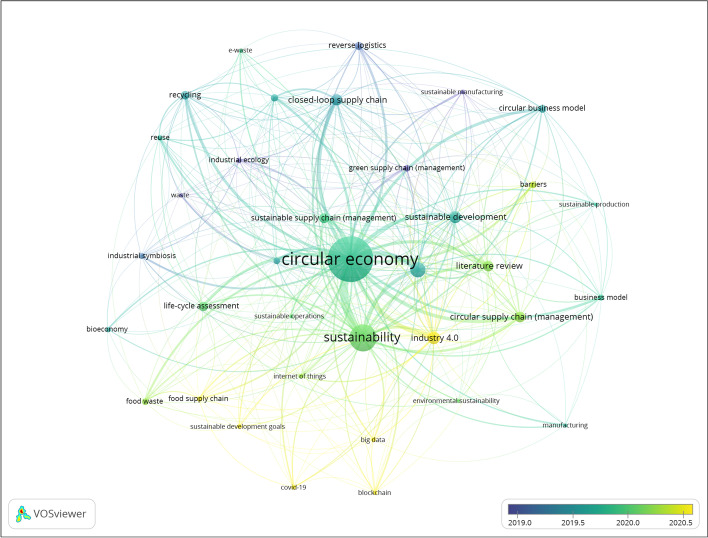
Visualization of co-occurrence of author keywords with temporal overlay (VOSviewer)

The VOSviewer settings were set to at least 15 co-occurrences per keyword, resulting in 36 mapped keywords overall. To avoid duplications in the visualization, synonymous terms or different variants of keywords were merged using a thesaurus file. The temporal co-occurrence map is depicted in Fig. 4. The most used keywords appear in the largest circles, and the strength of the co-occurrence between two keywords is indicated by the strength of the combining link. Table 4 presents the list of the top 20 keywords, their absolute occurrence, and their link strength.

**Table 4:** Data on author keywords (top 20)

Rank	Keyword	Occurrence	Total link strength
1	circular economy	813	1277
2	sustainability	340	654
3	supply chain (management)	135	296
4	industry 4.0	98	213
5	literature review	71	178
6	sustainable development	87	178
7	closed-loop supply chain	80	176
8	circular supply chain (management)	77	153
9	sustainable supply chain (management)	69	118
10	recycling	56	113
11	circular business model	46	112
12	remanufacturing	43	93
13	life-cycle assessment	59	91
14	business model	32	77
15	reverse logistics	47	77
16	barriers	33	76
17	waste management	36	72
18	reuse	26	65
19	industrial ecology	26	62
20	food waste	37	60

The most common keywords retrieved were — unsurprisingly, as the map was created based on these search terms — CE, sustainability (and sustainable development), and supply chain (management). Setting aside these keywords, the next most frequently studied topics were industry 4.0; literature review; sustainable development and three different supply chain management frameworks, namely, closed-loop, circular, and sustainable. The only methodological keyword within the top 20 was “literature review” (R5). This underscores the early research stage of the CSC framework and its theoretical and conceptual nature.

**Fig. 5:**
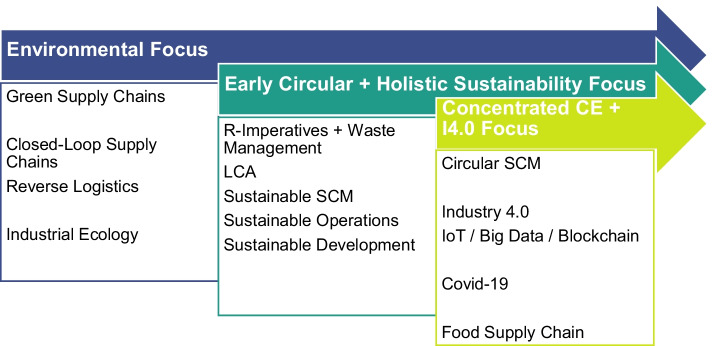
Thematic clusters in the evolving CSC research field

As the colors in the map do not represent thematic similarity but, rather, how recently the keywords appeared in the body of literature [58], topics of current interest or so-called hot topics within the field can be identified (Fig. 5). Three main temporal clusters can be identified: starting with an emphasis on environmental issues, the research concentration shifted toward an early circular and more holistic sustainability focus before the CSCM research field emerged as a separate one. While the earliest cluster still features concepts such as green supply chain management — which shows an average publication year of 2016.35 — the second cluster focuses more on R-imperatives such as recycling (R10) or reuse (R18) and sustainability-related concepts. Most recently, new approaches concerning the Fourth Industrial Revolution (I4.0) and other digitally enabled developments, such as the Internet of Things, big data, and blockchain, have arisen in the body of literature. A current application of the circular supply chain research field is the food supply chain (R20; average publication year 2020.76). Publications focus on issues such as waste flows [59], food redistribution [60] and knowledge sharing and the impact of technology [61]. The most recent development within the research field is — also unsurprisingly — the COVID-19 pandemic. With an average publication year of 2021.06 and 19 overall occurrences, the influence of the global pandemic can be recognized within the CSCM research field (see, e.g., [62]).

### Qualitative Results

This section explains the results of the second part of the systematic literature review and presents the qualitative results obtained from the content analysis. These thematic results are divided into two parts. The first part reviews the final sample of research articles for explicit definitions on the terms CSC and/or CSCM and gives an answer to RQ2: What are the definitions, conceptualizations, and understandings within the research field? Based on an analysis of those findings, six CSC archetypal elements are proposed that synthesize the framework’s current understanding in the scientific literature. The second part includes a detailed discussion on the CSC framework’s relationships to other, traditional, and sustainability-related, supply chain frameworks and thus answers RQ3: What makes the CSC concept unique, and thus, what differentiates it from other concepts? Four propositions on the CSC are formulated to position this framework in the sustainability management research.

### CSC Definitions and Archetypal Characteristics

To provide a valid view of the current understanding of CSCs in the scientific discourse, the final sample was reviewed for explicit definitions of the terms CSC and/or CSCM. Out of the 127 articles from the final sample, 17 mention (explicit) definitions and thus comprise the important partial sample for the thematic analysis. Table 5 depicts these 17 definitions along with details on the article types and their research focus.

**Table 5:** Research focus and definitions of CSCs (partial sample)

No	Author	Article type	Research focus	Definition of CSC
1	Genovese et al. [63]	Literature review + case study	• To align SSCM and the CE concept • To analyze environmental implications of circularity compared to linearity	Circular supply chains provide the benefit of diverting used products from being discarded as waste through the recovery of value and reused in production of secondary products. [p. 353]
2	Masi, Day, Godsell [16]	Systematic literature review	• To synthesize goals and assumptions about CE on the meso-level (SC) • To assess the state of knowledge on SC configurations within the CE	[…] Circular Supply Chains (CSCs) that continuously sustain the circulation of value by combining the variety of possible CE SC configurations and are fully aligned with the principles of the CE are largely absent in practice and academia; instead current knowledge on SCs in the CE is fragmented between several fast-growing research streams. [p. 2]
3	Nasir et al. [64]	Literature review + LCA analysis	• To assess the environmental impacts associated with linear and circular SC • To understand dynamics and implications of circular systems	At a micro-level, the implementation of circular economy practices would push to the design of circular or reverse supply chains, enabling products at the end of their life cycle to re-enter the supply chain as a production input through recycling, re-usage or remanufacturing. [p. 444]
4	Angelis, Howard, Miemczyk [17]	Systematic literature review	• To examine the link between traditional SCM, SSCM and the CE • To discuss the implications for SCM in CSCs	[…] here we analyse the implications for the development of CSCs defined as the embodiment of CE principles within supply chains. [p. 429] + 5 propositions on the CSC
5	Batista et al. [2]	Content-based systematic literature review	• To progress existing SSC research in the light of CE • To understand the CSC phenomenon and different forms of CSCs	These fundamental propositions help us to specify a definition of circular supply chain, as follows: the coordinated forward and reverse supply chains via purposeful business ecosystem integration for value creation from products/services, by-products and useful waste flows through prolonged life cycles that improve the economic, social and environmental sustainability of organisations. [p. 446]
6	Geissdoerfer et al. [18]	Literature review + case studies	• To discuss sustainability performance of circular business models and CSCs • To propose a framework that integrates circular business models and CSCM towards sustainable development	We use the term circular supply chain management (CSCM), which comprises the configuration and coordination of the supply chain to close, narrow, slow, intensify and dematerialise resource loops. [p. 713] […] we define Circular Supply Chain Management (CSCM) as the configuration and coordination of the organisational functions marketing, sales, R&D, production, logistics, IT, finance, and customer service within and across business units and organizations to close, slow, intensify, narrow, and dematerialise material and energy loops to minimise resource input into and was and emission leakage out of the system, improve its operative effectiveness and efficiency and generate competitive advantages. [p. 715]
7	Leising, Quist, Bocken [65]	Case study	• To address new ways of supply chain collaboration that contribute to the transition toward CE in the Dutch building sector	[…] we define CE in supply chain collaboration as connecting a network of actors in their supply chain by managing data transparency, material flows and exchanges, responsibilities, predictability and sharing benefits. This goes beyond the concept of reverse and closed loop supply chains […] by taking a strategic perspective on the new role of organizations to redevelop supply chains through collaboration to close and to slow down resource loops. [p. 977]
8	Mangla et al. [66]	Literature review + expert feedback	• To identify key barriers to CSCM implementation • To examine the relationship between barriers and understand dynamics	A circular supply chain (CSC) represents to a restorative production system, where resources, enter an infinite loop of reuse, remanufacturing and recycling. [p. 551]
9	Mishra, Hopkinson, Tidridge [67]	Case study	• To understand how CE led closed-loop SC create value • To identify key challenges in implementing CSCs	The paper proposes the term ‘circular supply chain’ for cases where circular economy principles are explicitly incorporated in CLSC [closed-loop supply chains] for value creation. [p. 1]
10	Vlajic, Mijailovic, Bogdanova [68]	(Empirical) case study	• To develop theory on CSCs • To define, analyze and discuss value recovery processes and circular flows	We define a circular supply chain as a connected network of organisations involved in the design and management of value-adding processes and value recovery of a product, component or material. Recovered value can be seen in term of the economic, social and environmental benefits for these organisations and associated stakeholders. [p. 523]
11	Yang et al.Yang et al. [69]	Case study	• To explore the relationship between business model innovation and circularity in supply chains • To investigate the link between product-service systems and CSCs	[…] circular supply chains should have the following characteristics: (1) the inner cycles are prioritised over outer ones (e.g. reuse and recover comes before recycling) (2) slowing the cycles (e.g. using resources as long as possible) (3) reducing waste at every stage of the product life cycle (4) reduce, reuse, recycle and recover resources as much as possible [p. 499]
12	Farooque et al.Farooque et al. [3]	Structured literature review	• To conceptualize a new definition of CSCM • To map current state of research • To identify directions for future research	Circular supply chain management is the integration of circular thinking into the management of the supply chain and its surrounding industrial and natural ecosystems. It systematically restores technical materials and regenerates biological materials toward a zero-waste vision through system-wide innovation in business models and supply chain functions from product/service design to end-of-life and waste management, involving all stakeholders in a product/service lifecycle including parts/product manufacturers, service providers, consumers and users. [p. 884]
13	González-Sánchez et al. [7]	Systematic literature review	• To define and differentiate the term CSC • To identify underlying theories and dimensions for designing CSCs	Circular supply chains is a step beyond closed supply chains and green supply chains. Firstly, it expands the number of actors in the chain by also considering sectors other than that of origin. Secondly, the relationships between actors also change. [p. 7] Circular supply chain management (CSCM) offers a compelling perspective that included the vision of a zero-waste economy and the restorative and regenerative cycles designed based on circular thinking. [p. 7]
14	Hussain, Malik [15]	Systematic literature review	• To analyze the organizational enablers of CSCs and how they affect the environmental performance of SCs	We build on this stream of literature to posit that CSCs are enabled by establishing a) collaboration within the supply chain network and reconfiguration of the supply chains for industrial symbiosis b) a persuasive organizational narrative that encapsulates a stronger orientation towards sustainability. [p. 3]
15	Jia et al. [70]	Systematic literature review	• To discover the present state of research concerning SSCM toward a CE	Besides CLSC [closed-loop supply chains], CSCs [circular supply chains] are also an open system that allows resources to flow between different supply networks, and within different technological and natural material loops. [p. 12]
16	Vegter, van Hillegersberg, Olthaar [71]	Systematic literature review	• To identify the processes that conceptualize a CSC • To identify performance objectives of a CSC	Circular supply chain management is the design and control of a network of organizations and end-users that strives for economic, environmental and social benefits by reducing, maintaining and recovering resources. [p. 12]
17	Vegter, van Hillegersberg, Olthaar [72]	Systematic literature review	• To analyze the current state of development of performance measurement for CSCM •To identify opportunities	The definition of CSCM used in the current paper is: the design and control of a network of organizations and end-users that strives for economic, environmental, and social benefits by reducing, maintaining, and recovering resources in restorative and regenerative cycles. [p. 4]

Although the majority of the partial sample are systematic or structured literature reviews that analyze the CSC(M), no comprehensive compilation of definitions of the term *circular supply chain (management)* was found. Although the work of González-Sánchez et al. [7] is the exception as it includes a collection of definitions on the CSC, it also covers other, related supply chain concepts and focuses more on the configurations and enablers of the CSC rather than on the conceptualization behind each definition. Table 5 shows that the conceptualization is wide-ranging, varying in detail, scope, and focus. While some scholars merely describe what makes a supply chain a circular one and what its main characteristics are, others aim at holistically defining the CSC as a novel and, more importantly, distinct framework. Geissdoerfer et al. [18] and González-Sánchez et al. [7] each provide a definition of the term CSC as well as CSCM. Farooque et al. [3] and Vegter, van Hillegersberg, Olthaar [71] define only the term CSCM. However, the definition provided by Farooque et al. provides a comprehensive integrated view with the aim of distinguishing it from other sustainability research frameworks. The remaining definitions focus, with varying degrees of detail, exclusively on the term supply chain.

One of the most important conclusions that can be drawn from this analysis is that it appears that new definitions — and thus new, and possibly different, conceptualizations of the CSC framework — are no longer being introduced to the research field. In fact, out of the 17 definitions found through this systematic search, eleven are from 2017 and 2018. In the last year (2021), only one new definition was added — the one from Vegter et al. [72] that builds on their previous conceptualized definition formulated in a publication from 2020 and is only supplemented by a restorative and regenerative characteristic. The thesis that there is no longer a major accumulation of knowledge is also supported by the fact that the most recent articles from 2021 and 2022 almost all build on existing definitions, which is that presented in Table 5. For example, the definition provided by Batista et al. [2] is cited in recent works such as [73, 74], and [60]. However, the definition and conceptualization of Farooque et al. [3] is by far the most frequently cited and referenced (e.g., [75–79]); this suggests that the definition is the most comprehensive one as it captures the CSC as a differentiated concept.

A detailed analysis of the collected definitions, their further descriptions, and their detailed elaborations led to the extraction of key features. In this research paper, they are referred to as *CSC archetypal characteristics*. The identification of these characteristics followed the author-centric to concept-centric approach proposed by Webster, Watson [80]. This approach allows a synthetization of the literature by determining and discussing a framework’s key concepts or characteristics. It was further inspired by other sources, e.g., Stock, Boyer [81] and Ahi, Searcy [39]. Thus, it was possible to develop Table 6, which presents the six CSC archetypal characteristics resulting from the content-based literature review. These may be expressed as (1) R-imperatives, (2) restorative and regenerative cycles, (3) sustainability framework, (4) value focus, (5) holistic system-thinking, and (6) paradigm shift. These archetypal characteristics can be understood as distinct and unique features of the CSC concept that provide transparency for the CSC conceptualization. Too often, the term CSC means different things to different researchers and practitioners, and therefore, these characteristics aim to synthesize the various CSC properties. Table 6 presents them and their corresponding descriptions and contributing authors.

**Table 6:** CSC archetypal characteristics

No	CSC archetypal characteristics	Descriptions	Author contribution
1	R-Imperatives	A CSC systematically implements a waste hierarchy and circular retention strategies. These can be distinguished according to three loops: • short loops: refuse, reduce, reuse, repair • medium long loops: refurbish, remanufacture, repurpose • long loops: recycle, recover, re-mine	Ghisellini, Cialani, Ulgiati [47], Genovese et al. [63], Kirchherr, Reike, Hekkert [10], Nasir et al. [64], Angelis, Howard, Miemczyk [17], Batista et al. [2], Mangla et al. [66], Mishra, Hopkinson, Tidridge [67], Reike, Vermeulen, Witjes [13], Yang et al. [69], Farooque et al. [3], Bauwens, Hekkert, Kirchherr [82], Hussain, Malik [15], Vegter, van Hillegersberg, Olthaar [71]
2	Restorative and Regenerative Cycles	Within a CSC, resources are recovered through different cycles and circular flows. A key differentiation is made between • restorative cycles for technical products (not bio-organic materials) and • regenerative cycles for biological products (bio-organic nature) Another differentiation can be made between • closed-loop flows (= reverse-directed recovery flows) and • open-loop flows (= cascading forward-directed flows)	Ellen MacArthur Foundation [83], Ellen MacArthur Foundation [8], Geissdoerfer et al. [9], Masi, Day, Godsell [16], Angelis, Howard, Miemczyk [17], Batista et al. [2], Geissdoerfer et al. [18], Howard, Hopkinson, Miemczyk [84], Mangla et al. [66], Farooque et al. [3], González-Sánchez et al. [7], Hussain, Malik [15], Jia et al. [70], Morseletto [85], Vegter, van Hillegersberg, Olthaar [72], Zhang et al. [86]
3	Sustainability Framework	CSCs have a close but sometimes contested relationship to sustainability and sustainable development. The CE can at least be understood as a tool for sustainable development. Supply chains adopting CE principles could contribute to all three dimensions of sustainability such as • economic wins (e.g., reduced raw material and energy costs) • environmental wins (e.g., reduced waste and emissions) • social wins (e.g., new employment opportunities)	D'Amato et al. [87], Geissdoerfer et al. [9], Masi, Day, Godsell [16], Murray, Skene, Haynes [11], Angelis, Howard, Miemczyk [17], Batista et al. [2], Geissdoerfer et al. [18], Korhonen et al. [52], Korhonen, Honkasalo, Seppälä [88], Vlajic, Mijailovic, Bogdanova [68], Farooque et al. [3], Schroeder, Anggraeni, Weber [89], Suárez-Eiroa et al. [54], Hussain, Malik [15], Vegter, van Hillegersberg, Olthaar [71], LENGYEL
4	Value Focus	In CSCs, value has the highest priority. All core supply chain activities aim at proposing, creating, delivering, and capturing value, following a value logic framework: • value proposition (e.g., long-lasting products, products as service) • value creation and delivery (e.g., implementation of R-processes, waste elimination, use of renewables) • value capture (e.g., reduced economic, environmental, and social costs)	Richardson [90], Ellen MacArthur Foundation [83], Ellen MacArthur Foundation [8], Genovese et al. [63], Masi, Day, Godsell [16], Murray, Skene, Haynes [11], Nasir et al. [64], Nußholz [91], Angelis, Howard, Miemczyk [17], Batista et al. [2], Geissdoerfer et al. [18], Manninen et al. [92], Mishra, Hopkinson, Tidridge [67], Vlajic, Mijailovic, Bogdanova [68], Yang et al. [69], Farooque et al. [3], Suárez-Eiroa et al. [54], Morseletto [93], Vegter, van Hillegersberg, Olthaar [71]
5	Holistic System-Thinking	To enable the transition toward a CSC, holistic systems-thinking that incorporates every stage of the supply chain — from production to consumption, from every input to every output — is necessary. Only if the complex links and various interconnections between all actors of the supply chain as well as all resulting consequences of every supply chain action are understood and considered can a fully CSC be realized	Ellen MacArthur Foundation [8], Angelis, Howard, Miemczyk [17], Batista et al. [2], Howard, Hopkinson, Miemczyk [84], Geissdoerfer et al. [18], Kalmykova, Sadagopan, Rosado [94], Leising, Quist, Bocken [65], Mishra, Hopkinson, Tidridge [67], Vlajic, Mijailovic, Bogdanova [68], Farooque et al. [3], Suárez-Eiroa et al. [54], González-Sánchez et al. [7], Hussain, Malik [15], Jia et al. [70], Vegter, van Hillegersberg, Olthaar [71]
6	Paradigm Shift	The transition from a linear supply chain to a CSC can be characterized as a major paradigm shift. The shift from linear to circular requires a holistic transformation of economic, environmental, and corporate activities at all stages of the supply chain. To fully adopt CE principles, a paradigmatic, systemic, and thus high level of change in production as well as in consumption is needed	Loiseau et al. [95], Masi, Day, Godsell [16], Geissdoerfer et al. [9], Geissdoerfer et al. [18], Govindan, Hasanagic [96], Korhonen et al. [52], Vlajic, Mijailovic, Bogdanova [68], Farooque et al. [3], Hofmann [57], Hussain, Malik [15]

These characteristics were extracted to facilitate a clearer understanding of CSCs as a distinct concept within the sustainability management research field. In addition to the compilation of the definitions of the CSC, the identified archetypical characteristics provide a clearer profile of the CSC and thus help distinguish it more clearly from other frameworks such as SSCs and green supply chains. Only in this way will the newly emerging CSC concept have a chance of becoming permanently anchored in the sustainability management research field.

Table 7 shows the characteristics identified for each analyzed article in the partial sample. According to the analysis of these data, it is — again — evident that there is no total consensus between the 17 author groups and each of their CSC conceptualizations. The definition provided by Farooque et al. [3] (no. 12) is the most comprehensive one. While other definitions and research perspectives on CSCs lack some of the extracted archetypal elements, this definition comprehensively incorporates all of the identified main characteristics. Six definitions found within the literature review incorporate only three or less of the archetypal elements, which suggests that they do not provide as holistic or comprehensive an understanding as the others. There is some consensus among the authors regarding the archetypal characteristic *restorative and regenerative cycles*: 13 out of 17 articles (partially) consider this element, which demonstrates that it can be defined as a key characteristic for the CSC. The least-considered characteristic is *paradigm shift*: fewer than half of the analyzed contributions mention this element as an important feature of the CSC.

**Table 7:** Appearance of CSC archetypal characteristics (in partial sample)

CSC Definition # CSC Archetypal Characteristic	1	2	3	4	5	6	7	8	9	10	11	12	13	14	15	16	17	∑
R-imperatives	✔		✔	✔	✔			✔	✔		✔	✔	✔	✔		✔	✔	12
Restorative and Regenerative Cycles	✔	✔		✔	✔	✔	(✔)	✔	(✔)			✔	✔	✔	✔		✔	13
Sustainability Framework		✔			✔	✔			(✔)	✔		✔	(✔)	✔		✔	✔	10
Value Priorities	✔	✔	✔	✔	✔	✔			✔	✔	✔	✔				✔	✔	12
Holistic System-Thinking				✔	✔	✔	✔		✔	✔		✔	✔	✔	✔	✔	✔	12
Paradigm Shift		✔		✔		✔				✔		✔	(✔)	✔				7

While some allocations could be made directly from the definitions provided, some were made based on in-depth analysis of the research papers.

(✔) suggesting that some aspects of the characteristic are included while others not

It is possible that the lack of consensus among the authors can be explained by the absence of a holistic view of the supply chain in the CE context. This highlights the research gap that this article attempts to fill and confirms the purpose of this paper to facilitate the understanding of the CSC framework, including the collection of definitions on the concept as well as the clarification and categorization of archetypal characteristics. The latter, in particular, serves as the basis for answering RQ3, specifically, the differentiation of the CSC framework from other research concepts in sustainability management. In the following section, four CSC propositions and their associated categorizations are presented in detail.

### CSC Propositions

To answer RQ3 and the issue of what makes the CSC unique, specifically, what distinguishes the CSC concept from other sustainability concepts, this section presents four propositions to provide conceptual transparency for future CSC research. These four propositions are the result of the literature synthesis of the CSC framework and summarize the unique CSC characteristics, which clearly distinguish the CSC framework from related concepts.

#### Regeneration

As presented in “Conceptual Background” and “Qualitative Results”, the restorative and regenerative cycles constitute a distinctive attribute of the CE ideal. It was explained that there is a clear distinction between restoration and regeneration and their corresponding cycles. According to McDonough, Braungart [97], the planet on which we live has two different metabolisms: the biological (biosphere) and the technical (technosphere) cycle [97]. In the second cycle — the cycle of the industry — the technical nutrients (inorganic or synthesized materials such as metals or plastics) stay in a closed loop by applying restorative processes in such a way that they are returned to a previous, original or improved state [2, 85, 97]. Restoration can be achieved by repairing, refurbishing, remanufacturing, and recycling [83, 98].

While this restorative dimension is one that is commonly known from other sustainability-related concepts within the SCM field (e.g., in green or closed-loop supply chains [99]), it is the regenerative dimension that defines the CE and thus distinguishes the CSC from other frameworks [3, 100]. Although the term regeneration is not easily differentiated from the previous term of restoration [85], the two terms can and should be distinguished based on metabolism cycles: while recovery processes for technical nutrients take place within the technical cycle, regeneration for biological nutrients takes place within the biological cycle. A biological nutrient can be defined as “a material or product that is designed to return to the biological cycle — it is literally consumed by microorganisms in the soil and by other animals” [97]. Such materials are organic and therefore can decompose in the natural environment (e.g., water or soil) and provide food and balance for the ecosystem [97, 101]. Compared to technical materials and products, biological materials have the ability to become nutrients in the soil and other ecosystems in the biosphere, creating natural capital for reuse [102].

**Fig. 6:**
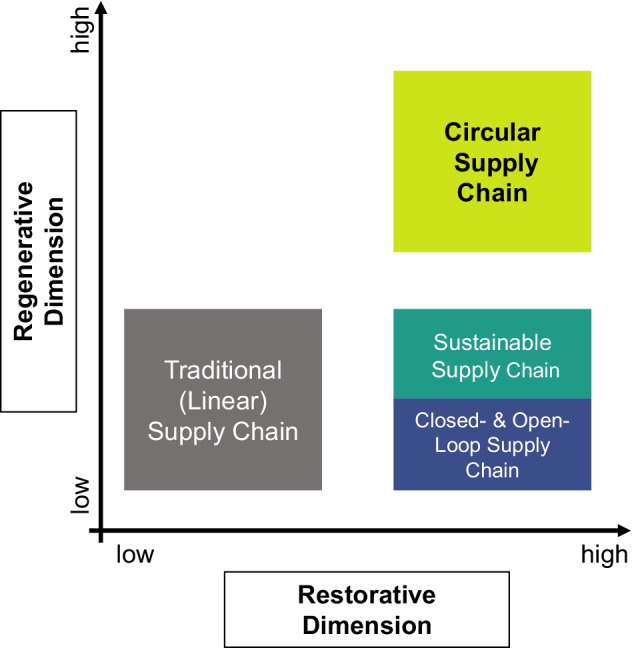
Restorative and regenerative dimensions of CSCs

Figure 6 illustrates the dimensions of restoration and regeneration with a four-field matrix, with either a low or high expression of the property. Although this separation is quite rigid and simple, it allows for a clear classification that furthers the understanding of the CSC, facilitating its differentiation from other sustainability management frameworks. Traditional — and mostly linear — supply chains typically incorporate only some or no restorative and regenerative flows at all. SSCs and closed- and open-loop supply chains, on the other hand, include restorative flows at their core (e.g., repairing, recycling, remanufacturing). However, CSCs set themselves apart from these frameworks by adding a regenerative dimension to their supply chain activities. Hence, the following proposition is suggested:

- **Proposition 1: Circular supply chains have both restorative and regenerative dimensions.**

#### Open Loops and Cascading Flows

Closely related to the previous dimension of regeneration is the open-loop and cascading dimension that represents a second important pillar, which is thus a distinguishing characteristic of the CSC. Within the well-established closed-loop supply chain, reverse directed recovery flows of material and products (such as reuse, refurbishing, remanufacturing, or recycling) are already a major supply chain activity [86, 103]. The direction of these flows is reversed and therefore in the opposite direction of the original, forwarded flow of materials and products. In addition, these reverse flows remain — as the name already suggests — within the supply chain. More explicitly, reverse flows are beneficial, for example, by returning recovered packaging material to the producer within the supply chain for further value recovery [40].

In a CSC — in addition to the closed-loop recovery flow — there is another type of material flow. This flow is an open-loop, forwarded cascading flow that brings secondary material (such as used goods, parts, components, byproducts, and useful waste) to other organizations outside of the supply chain [2, 3, 83, 86]. These open-loop flows are advantageous because recovered material that cannot be further used within the supply chain substitutes for virgin material in other supply chains, leading to an overall reduction in waste and virgin material use [3, 98]. Furthermore, these open-loops aim toward the zero-waste ideal and can be directed to other supply chains within either the same or other sectors [86].

**Fig. 7:**
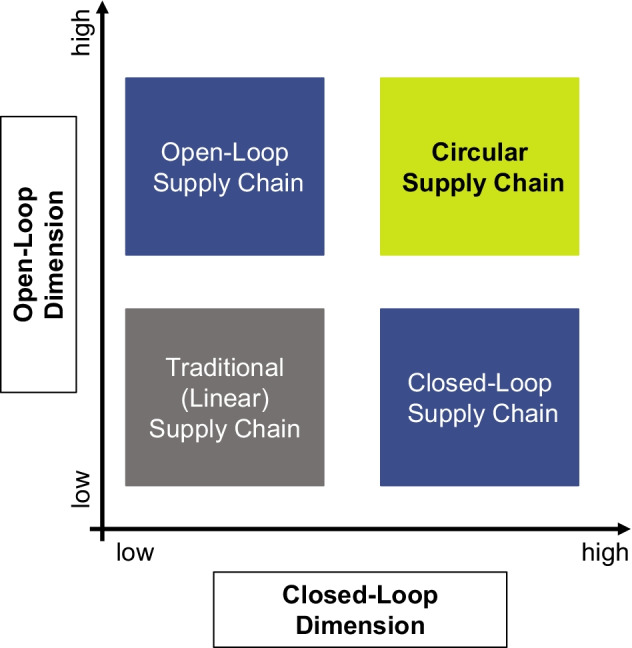
Closed- and open-loop dimensions of CSCs

It can therefore be concluded that the CSC extends the earlier sustainability management research frameworks via an open-loop and cascading flow dimension (see Fig. 7). Hence, the following proposition is suggested:

**Proposition 2: In a CSC, closed- and open-loop flows are applied with the goal of reducing waste and virgin material use within as well as across supply chains.**

#### Value Creation Focus

The integration of circular thinking into the production system changes the perspective on values and their potential for the supply chain [83]. The third fundamental proposition of the CSC is therefore one that was already mentioned within the archetypal characteristics of the CSC, specifically, the focus on values. By applying closed-loop as well as open-loop flows within and across the CSC, one major focus is on value creation. In particular, the supply chain is a key force for value creation because circular flows are the core of the CSC [18, 68].

The Ellen MacArthur Foundation developed four key principles of value creation that are fundamental to the CE ideal: the power of the inner circle, the power of circling longer, the power of cascaded use, and the power of pure inputs [8]. The inner circle refers to repairing or maintaining a product rather than remanufacturing or recycling it because more of the original value is preserved. The closer the circle in which a product flows, the more value is maintained [8]. Reike et al. conceptualized the closely connected R-imperatives and their preferred application within the supply chain. The lower the number associated with the imperative, the greater the focus on this imperative should be (e.g., R3 = repair preserves more value than R6 = repurpose where discarded goods or components are adapted for a different use). Longer circles aim at increasing the number of consecutive cycles and/or the time in each cycle. Through reuse or actions that extend product life, the timing of the resource loop is slowed [8, 104]. A cascaded use aims at substituting inflows of virgin materials, which results in a reduction of overall virgin material use [8]. Cascading refers to the above-described forward open-loop flows of secondary materials that connect producers, firms, and organizations across other supply chains [2]. One last key principle is the power of pure inputs. Value creation requires a certain level of purity of material and a certain level of quality of products and components. With nontoxic, easily separable inputs and designs, further potential for value creation is generated [83].

All four principles have one goal that they pursue: to create value whenever and wherever possible. This leads to a reduction or even complete elimination of waste. The conventional linear take-make-waste approach is neither designed nor targeted at value recovery or value creation. While, in SSCs, value creation takes place to some extent (e.g., R-activities such as recycling or reuse) and negative environmental and social impacts of the supply chain are reduced, the linear design is not fundamentally changed, so waste is still produced. A full CSC, on the other hand, focuses on value creation. By comprehensively redesigning the supply chain, the outputs of the production process become nutrients for further value creation rather than waste for disposal.

**Fig. 8:**
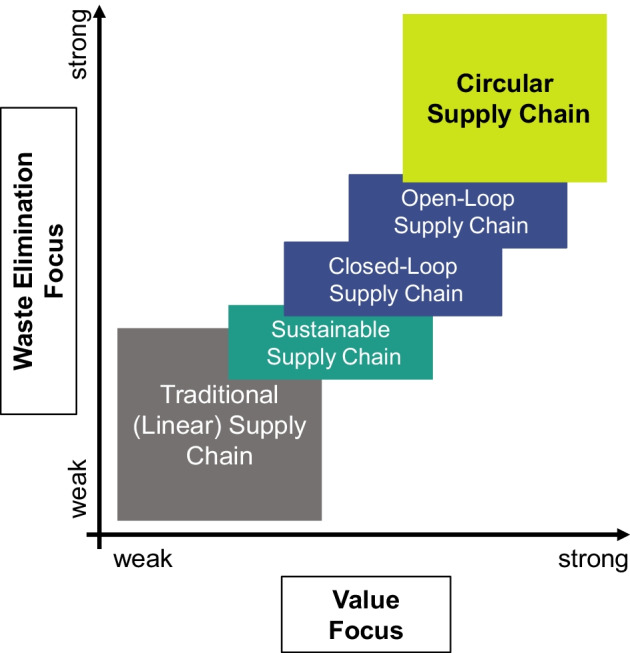
Value and waste elimination focus of CSCs

To distinguish the different supply chain concepts, Fig. 8 illustrates the intensity of value focus and waste elimination focus within different supply chain concepts. CSCs go beyond the boundary of related concepts and have a stronger focus on values and waste elimination [100]. The following proposition on the CSC is made:

- **Proposition 3: In CSCs, value creation and waste elimination are the core focus of all supply chain activities.**

#### Paradigm Shift

As described in the above sections, a transition toward a CE is associated with a major paradigm shift in production philosophy [2, 15, 52, 63]. Since supply chains are the key unit of action for the transition toward an ideal CE, the analysis of the adoption of CE principles into the supply chain is highly relevant.

**Fig. 9:**
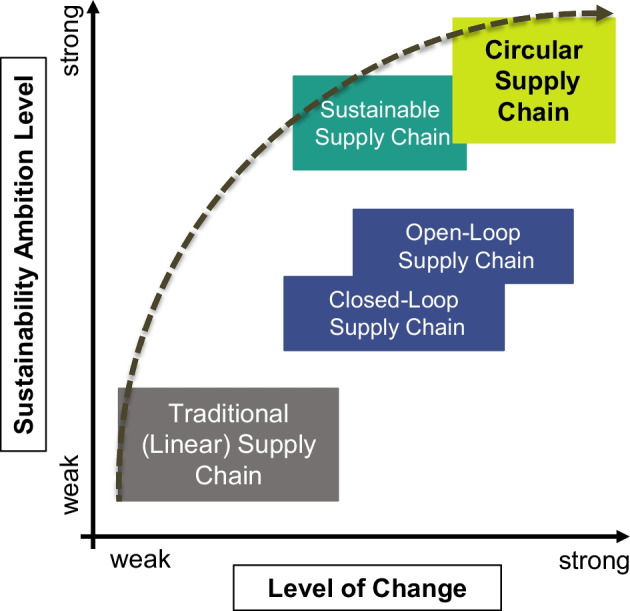
Level of change and sustainability ambition level of CSCs

Figure 9 links and synthesizes several findings and perspectives on the CSC framework, especially in research from Hussain, Malik [15], Geissdoerfer et al. [9], Reike, Vermeulen, Witjes [13], and Loiseau et al. [95]. Within this figure, different supply chain concepts are presented from a paradigm shift-sustainability-ambition perspective. Both perspectives can be synthesized from the previous three key propositions.

Since regeneration, open-loop, and cascading flows as well as a value creation focus are crucial principles of a supply chain in terms of a CE ideal, the supply chain’s organization and production in particular require major changes [7]. Angelis, Howard, Miemczyk [17] find that in the transition from linearity to circularity, major changes in terms of supply chain strategy, structure, flow, focus, scale, and scope are needed. While the authors draw on notions that have already been mentioned (e.g., closed and open material loops, technical and biological cycles), they reason that supply chain relationships and therefore organizations must change when moving toward a CSC (e.g., in terms of ownership or procurement). Hussain, Malik [15] and Geissdoerfer et al. [9] both argue that a radical change is needed, not only in how we produce goods and services but also in how we consume. In a CSC, consumers and especially their consumption behavior play a significant role in supply chain performance because consumers must be more actively integrated to successfully implement CE principles such as reuse and remanufacturing [6].

While in the sections above, linear, sustainable, and CSC were differentiated by various other dimensions, the distinction here is made directly with the sustainability ambition level incorporated in the different supply chain concepts. Although the relationship between CE and sustainability is still under discussion in the scientific community (see, e.g., Geissdoerfer et al. [9] and Korhonen et al. [52], the understanding in this paper follows the conceptualizations of Batista et al. [2], Farooque et al. [3], Hussain, Malik [15] and, most recently, Farooque et al. [100] that CSCs expand and enhance previous supply chain concepts (such as closed- and open-loop supply chains, green supply chains or SSC) in terms of sustainability orientation and sustainability ambition. However, CSCs distinguish themselves from other frameworks only if economic, environmental, and social goals are explicitly incorporated and aligned [18]. Only if the CSC concept strives to balance the sustainability dimensions will they have a transformative impact.

In summary, CSCs need major organizational, structural, and institutional change that is required to meet the challenges of sustainability in production and consumption [95]. Hence, the following and last proposition is suggested:

- **Proposition 4: CSCs require a paradigm shift and have a strong sustainability ambition level.**

## Conclusion

There is growing interest in the CSC, which is a trending concept in the field of sustainable business management. Within the last 5 years, the number of research articles on supply chains in light of the CE has increased significantly. As is so often the case when a new concept catches on, there is a risk that its meaning will become diffuse and the term itself will be used only as a buzzword. To mitigate that risk, the current paper has explored the CSC as a concept and has aimed to provide transparency for a better and more differentiated understanding of the CSC. Although at least ten CSC review articles have been published thus far, none comprehensively and systematically analyzes CSC conceptualization. The focus of this paper was to gather definitions of the CSC(M) term, synthesizing the existing literature to provide transparency for future research in this field. In the following subsections, theoretical and practical contributions are highlighted, and limitations and possible future research opportunities are presented.

### Theoretical Contribution

To date, this is the first attempt to comprehensively collect CSC definitions to further — and more importantly, holistically — conceptualize the framework within the sustainability management research field. A systematic literature review of the current research base revealed that the CSC framework, its current interpretation, and especially its relationship to other sustainability-related management frameworks have not been adequately addressed. This resulted in the following main research question:

- **What are the current understandings among scholars of the CSC concept and CSCM framework?**

In response to this research question, a systematic literature review was conducted. The two major databases, WOS and Scopus, were used to search for research articles that consider the CSC as a distinct concept and thus include new definitions, conceptualizations, theoretical, or applied frameworks and case studies on the CSC. Nearly 1500 articles were located and evaluated based on quality and content. After a careful screening process, 127 articles remained for further in-depth analysis.

The findings of this study add to the literature on supply chains in the context of the CE and make three major theoretical contributions. These can be best summarized through the proposed subquestions that were answered throughout the paper:

1. **What are the main research streams and core topics within the research field?**
  The first contribution is the identification of the main research streams and core topics. To identify the direction of development of the CSC research field, VOSviewer software was used to visualize the co-occurrence of author keywords in a temporal overlay. Three thematic clusters were identified: (1) a pure environmental focus, (2) an early circular and holistic sustainability focus, and (3) a more concentrated CE focus as well as a digitalization focus. In particular, the last and thus most recent cluster already incorporates the COVID-19 pandemic, which illustrates the timeliness of the research field. To date, this is the first attempt to identify and visualize the temporal evolution of the still young CSC research field.
2. **What are the definitions, conceptualizations, and understandings within the research field?**
  A second contribution is the systematic and comprehensive collection of CSC definitions and conceptualizations. Out of 127 reviewed articles, seventeen provide a distinct and clear definition of the CSC concept. Although a large number of the articles reviewed are systematic or structured literature reviews, no comprehensive compilation of definitions was found. This highlights a gap that this research paper aims to fill and confirms the purpose of this article in conceptualizing the CSC. Furthermore, the existing conceptualizations were found to be broad and vary in detail, scope, and focus. Thus, a further in-depth analysis of the identified sample led to the identification of six archetypal elements of the CSC: (1) R-imperatives, (2) restoration and regeneration cycles, (3) sustainability frameworks, (4) value focus, (5) holistic systems-thinking, and (6) paradigm shifts. These further add to the CSC literature and contribute to a more transparent understanding.
3. **What makes the CSC concept unique, and thus, what differentiates it from other concepts?**
  Last, a third contribution is the identification of four propositions on the CSC framework that offer novel and important insights into how CSCs differ from traditional (linear) supply chains, open- and closed-loop supply chains, and sustainable supply chains. In doing so, this paper contributes to the literature by providing clear statements on CSC uniqueness, creating much needed transparency for the research field and future research on this topic. The term CSC is multifaceted and means different things to different people. To overcome the framework’s ambiguity, this article has attempted to synthesize the CSC’s core properties: (1) regeneration, (2) open loops and cascading flows, (3) value creation focus, and (4) paradigm shift. These properties set the CSC apart from other sustainability-related supply chain concepts, and therefore, a clear and much-needed differentiation is provided.

Overall, this paper contributes to various calls from the research community to conceptualize the CSC and to identify the current state of the research (e.g., Angelis, Howard, Miemczyk [17] Batista et al. [2] and Farooque et al. [3]).

### Practical Contribution

Although the contributions of this paper are primarily theoretical in nature, some practical contributions can be concluded. These can be derived from the core properties of the CSC that were developed in “CSC Propositions”. First, the regenerative aspect — as well as the restorative aspect — cannot be neglected if managers want to change their supply chain activities from predominantly linear to circular processes. Regenerative cycles in which biological materials are recirculated must be designed and implemented efficiently. If supply chains are no longer designed to create waste but to create future input, core supply chain functions must adapt. In practice, this means, in particular, changing and adapting product development that aims from the outset to ensure that products can be easily returned to either the biological or technical cycle. Another important adjustment must be made in procurement, as it is no longer possible to procure products that are not suitable for circulation [100]. Second, as CSCs circulate not only in closed but also open- and forward-directed loops, CSC managers need to collaborate more intensely with other, often cross-sectional, supply chains to succeed in achieving the zero-waste ideal. Long-term circularity collaborations with other industries, businesses, and organizations are needed [78]. Third, in the transition toward a CSC, managers need to focus on implementing a systematic waste hierarchy. The holistic implementation of R-imperatives that cover the entire product lifecycle is key when aiming to close the (linear) supply chain gap. In a CSC, the core supply chain processes are supplemented by additional processes, increasing the complexity of the tasks for managers. In particular, the process “use” and thus the consumer come to the center of attention [71]. Finally, managers of a (soon-to-be) CSC face the consequences of a major paradigm shift if they truly and systematically work toward achieving a full CSC. Many challenges — organizational, governmental, or internal challenges from the supplier or consumer side — will arise during the process of CE implementation [96]. To be successful in the long term, the management of a CSC requires highly qualified and competent CE experts to support a paradigm shift toward circularity. In addition, full commitment from top-level management is needed.

### Limitations

The research presented here is not without limitations. The methodology of the literature review was systematic and enabled the identification of important contributions to the CSC research topic. The selection process itself focused on the terms CE and supply chains within titles, abstracts, and keywords and therefore may have been too rigorous, potentially overlooking other important contributions. In addition, the search focused only on the two major databases — WOS and Scopus — disregarding other sources of potential articles. The exclusion of book chapters and proceedings, as well as all non-English language works, also limits a more comprehensive analysis and further contributions to the conceptualization of the CSC term. However, these exclusions were intentional to ensure that the review focuses on academic articles within the CSC research field.

The focus of this paper has been to compile written definitions of the terms CSC and CSCM to understand the concepts and provide transparency so that they can be distinguished from other related frameworks. Nevertheless, a definition is often brief and rather narrow compared to the overall understanding. Thus, this approach may neglect some dimensions of the CSC as they are not present in the (most likely) narrow CSC definition [10]. However, it is still of academic relevance how a concept — in particular a trending one — is understood among scholars in the research field.

### Future Research Opportunities

An important finding of this paper is that the CSC framework is only at an early stage of research. The compiled definitions of the term CSC clearly demonstrate this, as they vary widely in scope, depth, and focus. The CSC field is rapidly evolving, so an update of the literature in the field will be needed in the coming years to capture the direction of development.

The CSC framework provides an interesting and promising research topic that aims to replace the end-of-life concept and accelerate the transition from linear to circular production and consumption. As this research article focused on the conceptual CSC understanding, future research opportunities lie in the practical adoption of circular practices in supply chains. Issues of particular importance are summarized in Table 8.

**Table 8:** Future research opportunities

No	Future research opportunities	References
1	Circular supply chain process performance measurement • To develop new and adapted performance measurement systems for CSCs • To develop performance measures that provide a clear distinction between circularity and sustainability • To develop a performance measurement system that supports decision-making in CSCM	[72, 75, 105, 106]
2	The role of Industry 4.0 and technology in CSCM • To analyze which I4.0 technologies support the transition toward circularity • To develop state-of-the-art machine learning algorithms, big data and blockchain technology to address issues of the CSC	[107–109]
3	Collaboration, relationship management and organizational culture in CSCs •To understand which barriers hinder (cross-sectoral) collaboration • To assess the SC’s internal capabilities to transition toward a CSC • To analyze which human factors contribute to the implementation of a CSC	[78, 110]
4	CSC indicators, measurement and maturity assessment • To identify indicators that are independent and develop a robust and effective circular index • To create sector/industry specific circular measurement systems • To develop circular maturity frameworks assessing the transition toward a CE and level of SC circularity	[111–113]
5	Case studies and real-world data on CSC implementation • To collect and build a base of empirical research on CE implementation at the supply chain level • To analyze the economic, environmental, and social impacts of CE implementation	[114–116]

## Data Availability

All relevant data and material that was used to conduct this research can be provided upon request.
